# Switching the mode of sucrose utilization by *Saccharomyces cerevisiae*

**DOI:** 10.1186/1475-2859-7-4

**Published:** 2008-02-27

**Authors:** Fernanda Badotti, Marcelo G Dário, Sergio L Alves, Maria Luiza A Cordioli, Luiz C Miletti, Pedro S de Araujo, Boris U Stambuk

**Affiliations:** 1 Departamento de Bioquímica, Centro de Ciências Biológicas, Universidade Federal de Santa Catarina, Florianópolis, SC 88040-900, Brazil; 2 Departamento de Bioquímica, Instituto de Química, Universidade de São Paulo, São Paulo, Brazil; 3 Departamento de Produção Animal e Alimentos, Centro de Ciências Agroveterinárias, Universidade do Estado de Santa Catarina, Lages, Brazil

## Abstract

**Background:**

Overflow metabolism is an undesirable characteristic of aerobic cultures of *Saccharomyces cerevisiae *during biomass-directed processes. It results from elevated sugar consumption rates that cause a high substrate conversion to ethanol and other bi-products, severely affecting cell physiology, bioprocess performance, and biomass yields. Fed-batch culture, where sucrose consumption rates are controlled by the external addition of sugar aiming at its low concentrations in the fermentor, is the classical bioprocessing alternative to prevent sugar fermentation by yeasts. However, fed-batch fermentations present drawbacks that could be overcome by simpler batch cultures at relatively high (e.g. 20 g/L) initial sugar concentrations. In this study, a *S. cerevisiae *strain lacking invertase activity was engineered to transport sucrose into the cells through a low-affinity and low-capacity sucrose-H^+ ^symport activity, and the growth kinetics and biomass yields on sucrose analyzed using simple batch cultures.

**Results:**

We have deleted from the genome of a *S. cerevisiae *strain lacking invertase the high-affinity sucrose-H^+ ^symporter encoded by the *AGT1 *gene. This strain could still grow efficiently on sucrose due to a low-affinity and low-capacity sucrose-H^+ ^symport activity mediated by the *MALx1 *maltose permeases, and its further intracellular hydrolysis by cytoplasmic maltases. Although sucrose consumption by this engineered yeast strain was slower than with the parental yeast strain, the cells grew efficiently on sucrose due to an increased respiration of the carbon source. Consequently, this engineered yeast strain produced less ethanol and 1.5 to 2 times more biomass when cultivated in simple batch mode using 20 g/L sucrose as the carbon source.

**Conclusion:**

Higher cell densities during batch cultures on 20 g/L sucrose were achieved by using a *S. cerevisiae *strain engineered in the sucrose uptake system. Such result was accomplished by effectively reducing sucrose uptake by the yeast cells, avoiding overflow metabolism, with the concomitant reduction in ethanol production. The use of this modified yeast strain in simpler batch culture mode can be a viable option to more complicated traditional sucrose-limited fed-batch cultures for biomass-directed processes of *S. cerevisiae*.

## Background

The yeast *Saccharomyces cerevisiae *has been known to humans for thousands of years and it is routinely used in many traditional biotechnological processes, including bread making and production of several alcoholic beverages. Consequently, it has been extensively studied and thus is considered a model system for the metabolic, molecular and genetic analysis of eukaryotic organisms. Due to its GRAS status, *S. cerevisiae *yeasts are also applied on a huge scale in biomass-directed processes, such as the production of baker's yeast, yeast extract and other food additives (vitamins, proteins, enzymes, and flavouring agents) [1], as well as for production of heterologous proteins (including vaccines and other therapeutic compounds), or even for engineering completely novel metabolic pathways leading to the biotechnological production of important pharmaceuticals [2-5]. The combination of the large knowledge of yeast physiology, together with the fact that the yeast genome has been fully sequenced, has resulted in the development of production strains with optimized properties [6,7].

However, it should be stressed out that most of the industrial applications of *S. cerevisiae *rely in its ability to efficiently ferment sugars, even under fully aerobic conditions [8]. Since low by-product formation and a high biomass yield on sugar are prerequisites for the economic viability of biomass-directed applications, the occurrence of alcoholic fermentation in such processes is highly undesirable as it will reduce the biomass yield [9]. Aerobic ethanol production by *S. cerevisiae *cultures occurs when the carbon flux through glycolysis exceeds the capacity of the tricarboxylic acids cycle to completely oxidize the pyruvate produced. Thus, fully respiratory metabolism only takes place during the utilization of low sugar concentrations and slow rates of sugar consumption and growth, and plenitude of oxygen. Indeed, this yeast has developed several sensing and signaling mechanisms in order to not only ensure efficient sugar uptake from the medium, but to also repress alternative carbon source utilization and respiration, thus favoring the production of ethanol [10-14]. Accordingly, high cell concentrations are rarely feasible in a simple batch mode, as the required high initial sugar concentration would result in the significant production of ethanol, which can accumulate to values as high as 50% of the supplied sugar.

Consequently, in order to maximize biomass yield *S. cerevisiae *yeast cell are cultivated in a fed-batch manner, in which a sugar-concentrated solution is fed into the bioreactor under a variety of control strategies. Usually, after a batch phase, an exponential feeding profile is applied to ensure optimal production and growth conditions, followed by a decline phase at the end of cultivation. To ensure optimal oxidative growth several approaches have been developed to control the feed rate at a level below the critical value, beyond which ethanol is produced and therefore the biomass yield decreases. Nevertheless, supplementary equipment, complex control systems and kinetic models are usually required to monitor on-line the fermentation process in order to provide small sugar concentrations to the yeast cells, avoiding ethanol production [15-17]. Other technical and physical limitations, such as time delays or measurement noise, sub-optimal stirring and oxygen transfer, as well as non-homogenous supply of nutrients, may result in both a decrease of the growth rate of the microorganism and/or overflow metabolism, and consequently decreases in biomass productivity [18,19]. Many of these problems could be overcome by culturing the cells in the simpler batch mode, as long as overflow metabolism and/or ethanol production is prevented.

Sucrose is by far the most abundant, cheap and important sugar in the industrial utilization of the yeast *S. cerevisiae*. More than half of the world's ethanol production relies on the efficient fermentation of sucrose-rich broths such as sugarcane juice and molasses, and these raw materials are also used for the production of baker's yeast, and for production of several distilled alcoholic beverages [20,21]. It is generally accepted that *S. cerevisiae *cells harbor an extracellular invertase (β-D-fructosidase), that hydrolyzes sucrose into glucose and fructose, which are transported into the cell by hexose transporters and metabolized through glycolysis. This enzyme has been a paradigm for the study of protein synthesis and regulation of gene expression. Invertase is encoded by one or several *SUC *genes (*SUC1 *to *SUC5 *and *SUC7*), *SUC2 *being the most common loci found in almost all *S. cerevisiae *strains, including in other closely related yeast species [22,23].

These *SUC *genes generate two different mRNAs: a larger transcript encoding an invertase with a signal sequence required for its secretion from the cell, and a shorter transcript lacking this signal sequence and thus coding for an intracellular form of the enzyme [24]. While the former mRNA is repressed by high concentrations of sucrose or its hydrolysis products (glucose and fructose), the intracellular invertase is expressed constitutively. Finally, it has recently become evident that efficient invertase expression requires low levels of glucose or fructose in the medium [25-27]. Despite significant improvements in our knowledge regarding the molecular mechanisms involved in the repression of *SUC *expression, the transcriptional activator of this gene is still unknown [28,29]. A further level of complexity is the fact that invertase levels at the yeast surface are poorly (or even inversely) correlated with the ability of the cells to ferment this sugar, especially at high sucrose concentrations [30-32]. Extracellular sucrose hydrolysis may even allow growth of other microorganisms, including contaminant yeasts lacking invertase [33]. Extracellular production of fructose also imposes several problems to the industrial process due to slower fructose utilization by *S. cerevisiae *cells [34], which may result in residual sugar at the end of the cultivation with consequent losses in productivity.

In this study, a poorly characterized pathway for sucrose utilization in *S. cerevisiae *was engineered in order to improve biomass-directed applications of this microorganism. Several reports have shown that the kinetics of cell growth on sucrose by this yeast can only fit a model in which its utilization is composed of the contributions from both the direct uptake of sucrose, and the uptake of its hydrolysis products into the cell [35-37]. The analysis of direct sucrose uptake by *S. cerevisiae *cells revealed the presence of an active sucrose-H^+ ^symport [38,39] which was shown to be mediated by two different transport systems: high-affinity (*K*_m_~7 mM) uptake mediated by the the *AGT1 *permease, while the *MALx1 *maltose transporters allow the active uptake of sucrose with low (*K*_m _> 100 mM) affinity [40,41]. The active uptake of sucrose would justify the existence of the constitutive intracellular invertase, although sucrose can also be hydrolyzed by other intracellular glycosidases, such as α-glucosidase (maltase), an enzyme with the same affinity and activity for sucrose and maltose [42]. Indeed, we have recently shown that yeast strains unable to ferment glucose or fructose due to the absence of hexose transport, or strains lacking invertase, can actively transport sucrose into the cells allowing efficient fermentation of this sugar [43,44]. In the present report we show that when we modulate at the molecular level the rate of active sucrose uptake, we obtain yeast strains that can easily attain higher cell densities when grown in simple batch cultures with 20 g/L sucrose as carbon source. This novel and value strategy that improves one of the industrial applications of *S. cerevisiae *represents an interesting alternative to classical bioprocessing approaches.

## Results

### Kinetics of active H^+^-sucrose uptake in yeast

**Figure 1:**
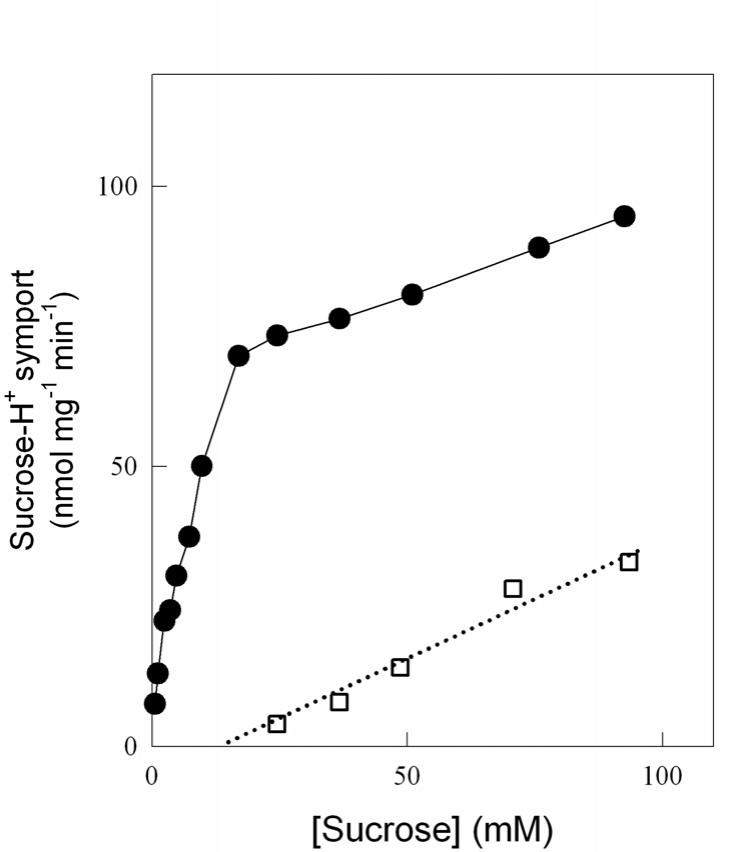
**Kinetics of active H^+^-sucrose symport activity in yeast strains**. The initial rates of active H^+ ^co-transport with sucrose were determined in yeast cells from strain 1403-7A (solid circles), or its *agt1*Δ counterpart strain LCM001 (hollow squares), pre-grown in rich YP media containing 20 g/L sucrose.

**Figure 2:**
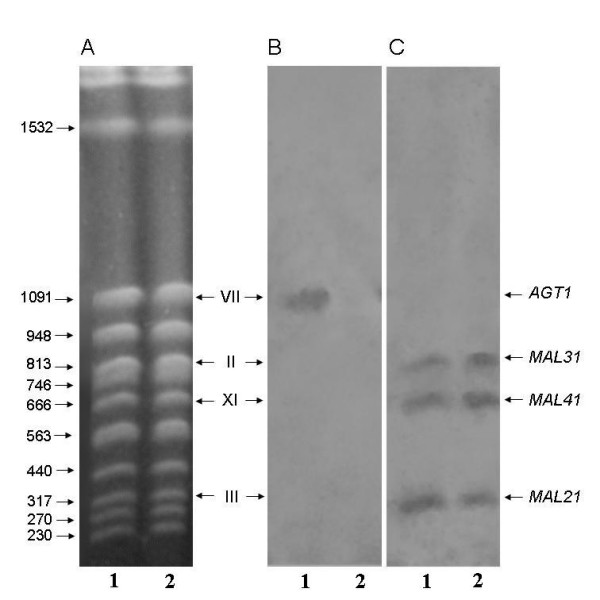
**Detection of active sucrose transporter genes in yeast strains**. Separation of chromosomes of yeast strains by PFGE and staining with ethidium bromide (**A**), and detection of sucrose transporter genes after chromosomes were blotted onto a nylon membrane and hybridized with a probe for *AGT1 *(**B**) or *MALx1 *(**C**). Lanes 1, strain 1403-7A; lanes 2, the *agt1*Δ strain LCM001. The values on the right in panel A are the sizes (in kilobase pairs) of selected chromosomes, the Roman numerals between panels A and B are chromosome numbers, and the positions of chromosomes carrying *AGT1*, *MAL21*, *MAL31 *and *MAL41 *are indicated on the right of panel C. Both chromosome sizes and numbers, and locations of transporter genes, were based on results obtained with the reference strains S288C and MC966A (see Table 1, data not shown).

Strain 1403-7A is a *MAL *constitutive strain that lacks invertase activity, but it still grows and ferments efficiently sucrose due to its active transport into the cell, and intracellular hydrolysis by a cytoplasmic α-glucosidase [42,44,45]. Since several different permeases (with differing kinetic properties) have been described as capable of active sucrose-H^+ ^symport [40], we decided to investigate the role that sugar transport could have on sucrose utilization by this yeast cells. Sucrose transport kinetics by strain 1403-7A (Figure 1) indeed indicated the presence of both a high-affinity (*K*_m_~7 mM) and low-affinity (*K*_m_~120 mM) transport activities, as has already been described for other wild-type yeast strains [40]. The *AGT1 *permease is responsible for the high-affinity component, while the low-affinity transport activity is mediated by any of the *MALx1 *maltose permeases. Indeed, this strain contains in its genome (Figure 2) at least 3 different *MALx1 *permeases, *MAL21 *(chromosome III), *MAL31 *(chromosome II), and *MAL41 *(chromosome XI), while the *AGT1 *gene is located in chromosome VII. It should be stressed out that both genetic and molecular studies [46,47] have already shown that in strain 1403-7A the *MAL31*, *MAL41 *and *AGT1 *permeases are functional, and we do not know if the *MAL21 *gene encodes for a functional permease. Our PFGE and blotting analysis also showed the presence of a unique *SUC *gene (*SUC2 *in chromosome IX, data not shown) in the genome of strain 1403-7A, probably a mutant *suc2 *allele of the gene since this strain lacks invertase activity [42,44,45].

In order to develop a new yeast strain that would take up sucrose from the medium by just the low-affinity transport activity, we deleted from strain's 1403-7A genome the high-affinity sucrose-H^+ ^symporter encoded by the *AGT1 *gene (see strain LCM001 in Fig. 2). As expected, the kinetics of active sucrose transport indicated that in the *agt1*Δ strain LCM001 sucrose-H^+ ^symport was mediated by a low-affinity and low-capacity transport activity (Fig. 1). Since the *AGT1 *permease is a low-affinity (*K*_m _20–30 mM) maltose transporter [41], in strain LCM001 maltose uptake from the medium was normally mediated by the above indicated high-affinity *MALx1 *(*MAL21*, *MAL31 *and *MAL41*) maltose transport activities (data not shown).

### Sucrose utilization by yeast strains

**Figure 3:**
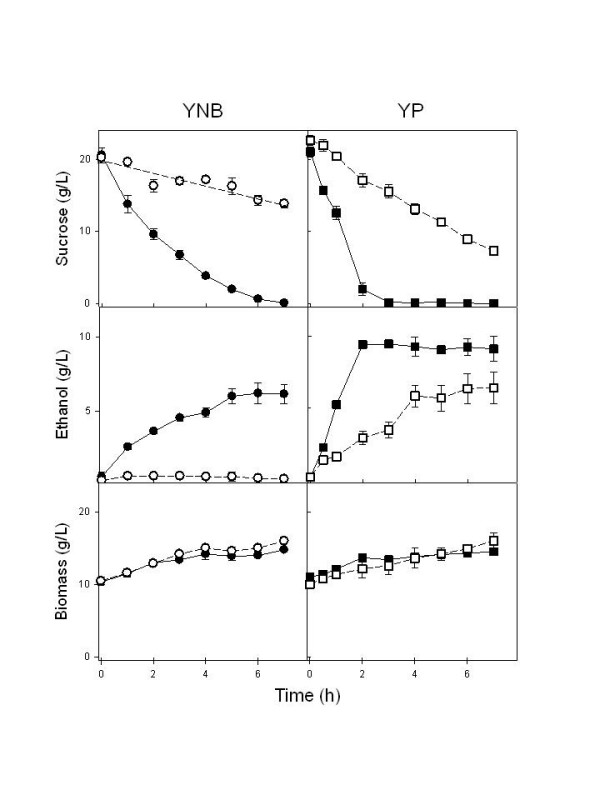
**Batch fermentations of 20 g/L sucrose by yeast strains**. The consumption of sucrose, and ethanol and biomass production during sucrose batch fermentations with 10 g/L yeast cells (dry weight) of strain 1403-7A (black symbols) or strain LCM001 (open symbols), were determined using synthetic YNB (circles) or rich YP (squares) medium. Data shown are means (± range) from two independent experiments.

**Figure 4:**
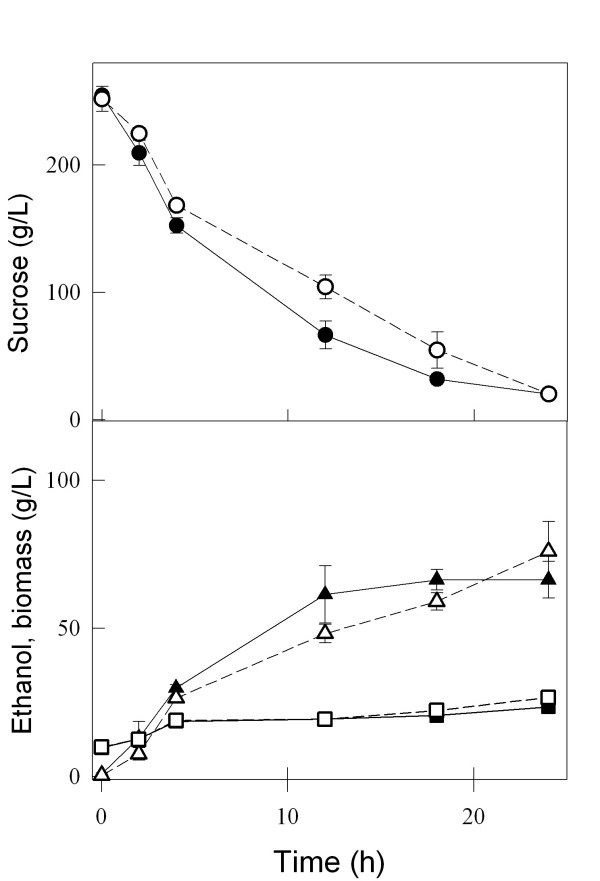
**Batch fermentations of 250 g/L sucrose by yeast strains**. The consumption of sucrose (circles), and ethanol (triangles) and biomass (squares) production during sucrose batch fermentations with 10 g/L yeast cells (dry weight) of strain 1403-7A (black symbols) or strain LCM001 (open symbols) were determined with rich YP medium. Data shown are means (± range) from two independent experiments.

The kinetics of sucrose consumption and ethanol production during batch fermentations (Figure 3) indicated that the *agt1*Δ strain LCM001 had a significant slower capacity to consume sucrose from the medium (at a concentration of 20 g/L), producing less ethanol than the parental wild-type strain 1403-7A. This phenotype was more pronounced when fermentations were performed in synthetic medium, compared with rich YP medium (Fig. 3). Strain 1403-7A showed rates of maximum sucrose consumption of 0.25 and 0.78 g sucrose (g cell dry weight)^-1 ^h^-1 ^in synthetic and rich medium, respectively, while the maximum sucrose consumption rate was reduced fourfold in the *agt1*Δ LCM001 strain, to 0.06 and 0.17 g sucrose (g cell dry weight)^-1 ^h^-1 ^in synthetic and rich medium, respectively. The beneficial effect of complex nitrogen sources (peptides or amino acids) compared with ammonium sulfate present in the synthetic medium has been described previously [48-50]. Furthermore, no major difference in the rates of sucrose consumption or ethanol production were observed among both strains when high sucrose concentrations (> 200 g/L) were used (Figure 4). The maximum sucrose consumption rates for the wild-type and the *agt1*Δ strains were 0.99 and 0.78 g sucrose (g cell dry weight)^-1 ^h^-1^, respectively, which is in accordance with the kinetics of active sucrose transport presented by each strain (Fig. 1). At 20 g/L sucrose (~58 mM) the low affinity transport system present in strain LCM001 is far from saturated (Fig. 1), while at this concentration the wild type strain's high-affinity transporter is operating with high capacity. At concentrations of ~0.73 M sucrose (250 g/L), probably all transport activities present in both strains are operating with high capacity allowing efficient sucrose fermentation. An intriguing observation was that even when the yeast cells were consuming sucrose more slowly, the increases in biomass during these batch fermentations were the same for both strains (Fig. 3 and 4). This result indicated that strain LCM001 is preferring to respire more of the carbon source (sucrose), and not fermenting it into ethanol.

**Figure 5:**
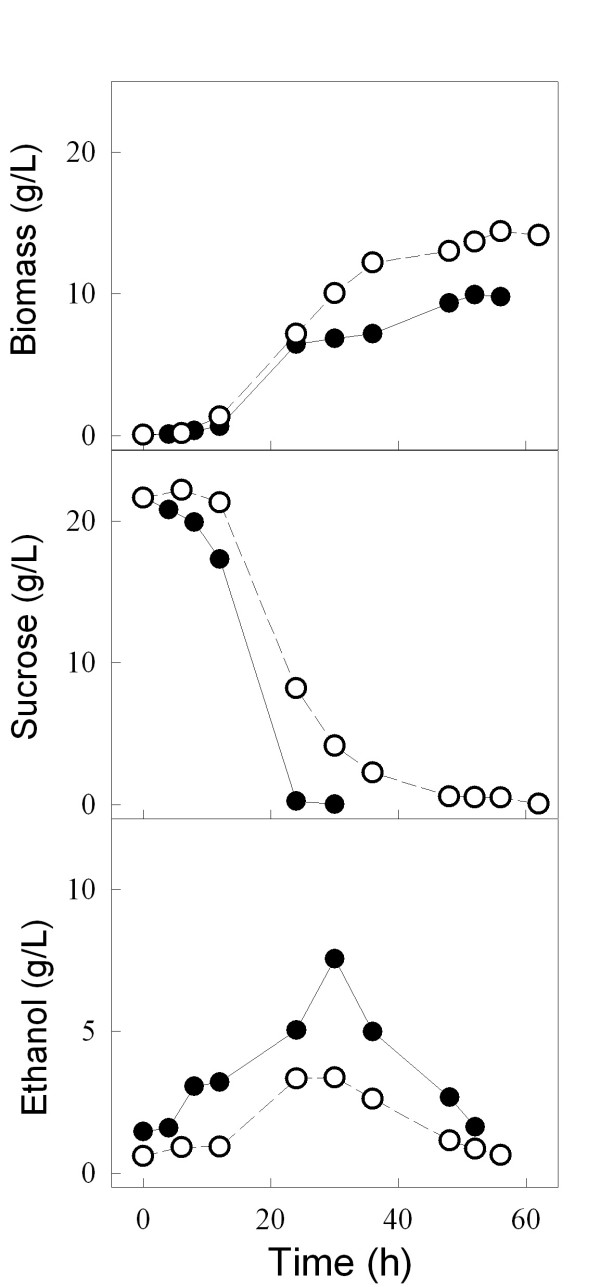
**Batch growth of yeast strains on 20 g/L sucrose**. The consumption of sucrose and the ethanol and biomass production by strain 1403-7A (solid circles) or strain LCM001 (hollow circles) were determined during growth in rich YP medium. For each strain a representative growth curve is shown.

Indeed, when we analyzed the kinetics of growth on sucrose by both strains (Figure 5), strain 1407-7A clearly presented a typical diauxic growth curve [51], where initially cell growth is coupled to efficient sugar consumption and ethanol production, when the carbon source is exhausted cells stop growing and the diauxic shift takes place, allowing the further consumption of the ethanol produced during sugar fermentation. However, strain LCM001 had a quite different pattern of sucrose utilization (Fig. 5). This *agt1*Δ yeast grow on sucrose efficiently, with the same specific growth rate (μ = 0.24 ± 0.02 h^-1^) as strain 1403-7A (μ = 0.24 ± 0.01 h^-1^) or other *SUC*^+ ^strains [44], although sugar consumption was slightly slower than with the wild-type strain (0.26 and 0.51 g sucrose [g cell dry weight]^-1 ^h^-1 ^for strains LCM001 and 1403-7A, respectively). These LCM001 yeast cells did not enter into the diauxic shift (Fig. 5), but continued to grow efficiently through time, reaching higher cell densities than the parental strain 1403-7A. Although it is expected that these batch cultures become oxygen limited during exponential cell growth, favoring sugar fermentation, strain LCM001 not only produced less ethanol from sucrose, but also started to consume the ethanol produced during growth even when significant amounts of sugar (~40% of initial value) still remained in the medium (Fig. 5). These results indicated that strain LCM001 had shifted its metabolism into a more aerobic sucrose utilization pattern than the parental strain.

**Figure 6:**
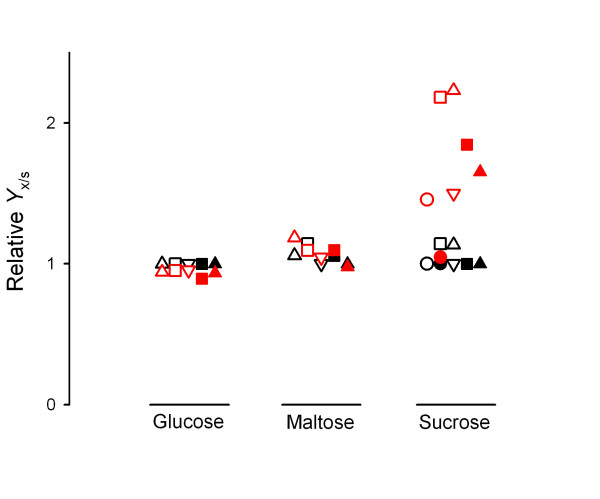
**Biomass yields by yeast strains**. The relative biomass yields, normalized to the values obtained by the wild-type 1403-7A strain in medium containing 20 g/L glucose, were determined during growth of strain 1403-7A (black symbols) or strain LCM001 (red symbols) in synthetic yeast nitrogen medium containing 5 (triangles), 10 (squares) or 15 (inverted triangles) g/L ammonium sulfate (open symbols) or peptone (close symbols) as nitrogen source, and 20 g/L of the indicated carbon sources. Results obtained with rich YP medium (circles) in the absence (open symbols) or presence (close symbols) of antimycin A are also shown. For the wild-type 1403-7A strain the *Y*_x/s _values varied between 0.20 ± 0.02 and 0.48 ± 0.03 g biomass (g glucose)^-1 ^when ammonium sulfate or peptone were used as nitrogen source, respectively, or 0.14 g biomass (g sucrose)^-1 ^when antimycin A was added to the medium.

We thus analyzed the biomass yield of these two strains during growth using three different carbon sources (glucose, maltose and sucrose) and different amounts (and quality) of nitrogen sources (Figure 6). It is evident that strain LCM001 produces from 1.5- to 2-fold more biomass during batch growth on sucrose (compared to the parental strain), while the biomass yields on glucose or maltose were unaffected. Conversely, while the ethanol yields on glucose by both the wild-type and the *agt1*Δ strain were similar and varied from *Y*_e/s _values between 0.16 ± 0.02 and 0.31 ± 0.03 g ethanol (g glucose)^-1 ^when ammonium sulfate or peptone were used as nitrogen source, respectively, the *agt1*Δ strain LCM001 produced only 10 to 40% of the ethanol produced by the wild-type strain 1403-7A when sucrose was used as carbon source. In order to confirm that this increase in biomass production by the LCM001 strain is due to respiration of the sugar, we added amtimycin A to the medium. Under this condition (respiration blocked), the biomass yield on sucrose of strain LCM001 was exactly the same as for the parental strain 1403-7A (Fig. 6), and both strains fermented sucrose efficiently (*Y*_e/s _= 0.50 ± 0.01 g ethanol [g sucrose]^-1^).

## Discussion

Due to the increased interest in biomass-based industrial applications of *S. cerevisiae*, several approaches have been developed to engineer the metabolism of this microorganism towards a more aerobic or respiratory utilization of sugars. In one approach where the targets are key regulatory proteins, either overexpression of the transcriptional factor *HAP4 *(activating respiratory genes), or deletion of *GCR1 *and *GCR2 *(activators of glycolytic genes), *HXK2 *or *REG1 *(relieving glucose repression), results in yeast strains showing increased biomass production during glucose fermentation [52-56]. However, modifying these regulatory circuits may also have some undesired side-effects, including significant reductions in the specific growth rate of the cells, and consequently losses in biomass productivity, or even altered patterns of sugar (other than glucose) utilization [57-61]. Another logic approach would be to restrict sugar uptake from the medium, which in the case of glucose transport by *S. cerevisiae *was a huge challenge as it was required not only to delete the hole set of hexose transporters found in this yeast (compromising almost 20 different genes), but also required the expression of a mutant chimera between the low-affinity (*HXT1*) and high-affinity (*HXT7*) glucose transporters as the unique sugar permease at the plasma membrane [62-64]. Although this yeast strain will respire and produce higher biomass levels when grown on glucose, it still has some inconveniences, such as slow growth rates, fructose fermentation, and an inability to use other sugars (e.g. galactose, due to *GAL2 *deletion). Indeed, the modification of the glucose uptake system in *E. coli *[65,66] also allowed the development of a bacteria with reduced overflow metabolism, and increased biomass production.

Our results show that when we engineer the mode of sucrose utilization by the yeast *S. cerevisiae*, allowing the direct uptake of the sugar by low-affinity and low-capacity transport systems, followed by its intracellular hydrolysis mediated by a maltase (α-glucosidase), the yeast cells grow efficiently on sucrose but produce significantly less ethanol since the cells are diverting more of this carbon source towards biomass production. Thus, higher biomass production can be attained with simple batch cultures in 20 g/L sucrose, avoiding some drawbacks of fed-batch cultures due to easier operational procedures, reduced equipment and process time needed for each production lot. Indeed, the more respiratory phenotype of strain LCM001 was clearly demonstrated by the occurrence of only one growth phase during batch cultivation with sucrose as the carbon and energy source, and the corresponding decrease (to the same levels of the parental strain 1403-7A) in the biomass yield when the respiratory inhibitor antimycin A was used.

Although *S. cerevisiae *has a strong tendency towards alcoholic fermentation of sugars, several reports have shown that in the case of some α-glucosides (e.g. maltotriose [67,68] and trehalose [69-72]) which are transported by low-affinity and/or low-capacity uptake systems, the sugar may be completely respired by the yeast cell. However, these approaches have little practical application due to the high commercial prices of these sugar substrates. Nevertheless, all these studies, including the results shown in the present report, highlight the importance of the sugar transport step in the aerobic/fermentative dissimilation of sugars by yeast cells [73]. It is also important to emphasize that the engineering strategy utilized in the present approach does not affect the utilization (and fermentation) of other sugars (e.g. glucose, fructose, maltose) commonly used by yeasts, and thus would not affect the downstream utilization of such strains in important industrial applications such as bread making, or production of distilled beverages. The high specific growth rates observed during batch growth of the engineered strain on sucrose, even when the sugar is consumed more slowly, is probably a consequence of the superior efficiency of this sugar (when compared to glucose) for binding to and stimulate the *GPR1 *sugar receptor in *S. cerevisiae *cells, an important signaling system that controls, among several other physiological aspects, yeast cell growth [10,12]. It would thus be interesting to delete this *GPR1 *sugar receptor, and analyze the consequence of such deletion on the growth rate and metabolism of sucrose by *S. cerevisiae *cells. Finally, another way to further improve the biomass yield of yeasts grown at excess sucrose concentrations could be obtained by combining the properties of strain LCM001 with one or more of the above described strategies (e.g. *HAP4 *overexpression, and/or *hxk2 *deletion), or even by using the classical fed-batch mode of yeast cultivation. It remains to be seen whether combination of such approaches can further improve the biomass yield of *S. cerevisiae *at higher sucrose concentrations.

## Conclusion

Higher cell densities during batch cultures on sucrose were achieved by using a *S. cerevisiae *strain engineered in the sucrose transport system. Deletion of the high-affinity sucrose transport system mediated by the *AGT1 *permease produced a yeast strain where sucrose was transported by low-affinity and low-capacity permeases. While up to 1.5 to 2-times more biomass, when compared with the parental strain, were obtained by the engineered yeast strain in simple batch cultivations using 20 g/L sucrose, the ability of the strains to efficiently ferment very-high sucrose concentrations (> 200 g/L) was unaffected. The yeast growth rate on rich medium containing 20 g/L sucrose was also unaffected, and thus the higher biomass yields were accomplished by preventing overflow metabolism and increasing respiration by the engineered strain, with the concomitant reduction in ethanol production. The simpler batch cultivation mode can be a viable option to more complicated traditional sucrose-limited fed-batch cultures. A thorough analysis of the physiological and transcriptional response of the engineered *S. cerevisiae *strain to very-high sucrose concentrations will help to better understand the regulatory mechanisms involved in sugar fermentation by yeasts, and could serve as a basis for engineering metabolic pathways to improve process performance of *S. cerevisiae *for biomass directed approaches using highly concentrated culture media.

## Methods

### Media and culture conditions

Cells were routinely grown on rich YP medium (10 g/L yeast extract and 20 g/L peptone), or synthetic medium (2 g/L yeast nitrogen base without aminoacids containing 75 mg/L L-tryptophane and 150 mg/L uracil) supplemented with different quantities of ammonium sulfate or peptone as nitrogen source, and 20 g/L glucose, sucrose or maltose as carbon source. The pH of each medium was adjusted to pH 5.0 with HCl, and media was either sterilized by filtration (synthetic medium), or autoclaved at 120°C for 20 min (rich YP medium). When required, 20 g/L agar, 3 mg/L antimycin A, or 200 mg/L geneticin (G-418) sulfate were added to the medium. Cells were grown aerobically at 28°C with shaking (160 rpm) in cotton plugged Erlenmeyer flasks filled to 1/5 of the volume with medium. The inoculum for growth assays was prepared by transferring aseptically a single colony from a plate into 5–10 mL of the selected medium containing 20 g/L glucose, and allowed to growth into stationary phase for 2 to 3 days before they were used to inoculate (by a 100 × dilution) new media containing the indicated carbon sources. Culture samples were harvested regularly, centrifuged (5,000 *g*, 1 min), and their supernatants used for the determination of sugars and ethanol. For batch fermentations yeasts were pregrown on YP-20 g/L sucrose until the exponential phase (~1 g of dry yeast/L), centrifuged (3,500 *g*, 3 min) and washed twice with cold water, and inoculated at a high cell density (10 g of dry yeast/L) into synthetic medium (4 g/L yeast nitrogen base without aminoacids and 10 g/L ammonium sulfate) or rich YP medium containing the indicated amounts of sucrose. Batch fermentations were incubated as described above for growth assays, and samples were collected regularly, centrifuged, and their supernatants analyzed as described below.

### Yeast strains

The *S. cerevisiae *strains and oligonucleotides used in the present study are described in Table 1. The yeast strains were kept at -80°C in 25% sterile glycerol, and from these frozen stocks yeast cells were streaked onto solid YP-2% glucose plates, incubated for 2 days at 28°C, and stored at 4°C (for a maximum of 1 month) until use. Standard methods for yeast transformation, DNA manipulation and analysis were employed [74]. The *AGT1 *gene was deleted according to the polymerase chain reaction (PCR)-based gene replacement procedure as described previously [43,50]. Briefly, the kanMX cassette from plasmid pFA6a-kanMX6 [75] was amplify with primers AGT1-pFA6-F1 and AGT1-pFA6-R1, and the resulting PCR product of 1,579-bp was used to transform competent yeast cells. After 2-hour cultivation on YP-20 g/L glucose, the transformed cells were plated on the same medium containing G-418 and incubated at 28°C. G-418-resistant isolates were tested for proper genomic integration of the kanMX cassette at the *AGT1 *locus by Southern analysis (see below) and analytical colony PCR using 3 primers (V-AGT1-F, V-AGT1-R and V-kanr-R; Table 1). These set of 3 primers amplified a 1,938-bp fragment from a normal *AGT1 *locus, or yielded an 813-bp fragment if the kanMX cassette was correctly integrated at this locus and replaced the *AGT1 *gene.

**Table 1:** *Saccharomyces cerevisiae *strains and oligonucleotides used in this study

	Relevant genotype or description:	Source or reference:
*Yeast Strains*		
1403-7A	*MAT***a ***MAL4*^*C*^*MGL3 suc*^-^*gal3 gal4 trp1 ura3*	ATTC 208023
LCM001	*agt1*Δ*::kanMX6 *derivative of 1403-7A	This study
S288C	*MAT*α*AGT1 MAL12 mal13 MAL31 MAL32 mal33 gal2 mel flo1 flo8-1 hap1 SUC2*	(39)
MC966A	*MAT***a ***MAL2 ura3-52 his3-11,15 leu2-3,112 SUC2*	(43)
*Primers*		
AGT1-pFA6-F1	AAGCAAGAAGAAGGCTGCCTCAAAAAATGAGGATAAAAACATTTCTGAGCGGATCCCCGGGTTAATTAA	Invitrogen
AGT1-pFA6-R1	AAAGGGATTCCTTATTTCTTCCAAAAAAAAAAAAACAACCCTTTTACTTAGAATTCGAGCTCGTTTAAAC	"
AGT1-F	AGGAGCTCATGAAAAATATCATTTCATTGG	Gibco BRL
AGT1-R	TTGGATCCACATTTATCAGCT GC	"
MALx1-F	CCATACTTGTTGTGAGTGG	"
MALx1-R	TCATTTGTTCACAACAGATG	Invitrogen
SUC2-F	GCGATAGACCTTTGGTCCAC	Gibco BRL
SUC2-R	GGACCGTGGTAACTCTAAGG	"
V-AGT1-F	GAATTTTCGGGTTGGTG	"
V-AGT1-R	TTGGATCCACATTTATCAGCTGC	"
V-kan^r^-R	GGAATCGAATGCAACCGG	"

### PFGE, chromosome blotting and hybridization

Yeast chromosomes were prepared as previously described [76] from 1 ml of yeast cells pre-grown in YP-2% glucose medium and collected at the stationary phase of growth. Cells were washed with 10 mM Tris-HCl, pH 7.5, containing 50 mM EDTA, and resuspended in the 0.4 ml of 4 mM Tris-HCl, pH 7.5, containing 95 mM EDTA, 130 mg/L of Zymolyase 20T and 7 g/L of molten (42°C) low-melting-point agarose. After solidification in a mold (Pharmacia Biotech), the agarose blocks were immersed in 10 mM Tris-HCl, pH 7.5, containing 0.5 M EDTA, and incubated at 37°C for 8 hours. Following a subsequent incubation in 10 mM Tris-HCl, pH 9.5, containing 0.5 M EDTA, 1% *N*-lauroylsarcosine, and 2 g/L proteinase K at 50°C overnight, the blocks were washed in 10 mM Tris-HCl, pH 7.5, containing 50 mM EDTA, and stored at 4°C in the same buffer. Each low-melting-point agarose block was transferred to a 10 g/L agarose gel in 50 mM Tris-HCl, pH 8.3, containing 50 mM boric acid and 1 mM EDTA. Pulsed field gel electrophoresis (PFGE) was performed at 10°C using a Gene Navigator pulsed-field system (Pharmacia Biotech) for a total of 27 hours at 200 V. The pulse time was stepped from 70 seconds after 15 hours to 120 seconds for 12 hours. Following electrophoresis, the gel was stained with ethidium bromide and photographed. The chromosomes separated by PFGE were transferred to a nylon filter (Biodyne A, Gibco BRL) by capillary blotting [74], and labeling of DNA probes (see below), including the pre-hybridization, hybridization, stringency washes and chemiluminescent signal generation and detection was performed by using a AlkPhos kit (GE Healthcare/Amersham Biosciences) as recommended by the manufacturer. After hybridization, an autoradiography film (Hiperfilm™ ECL – Kodak) was exposed to the membrane for 2 to 3 h before it was developed. Images were obtained by scanning with an ImageScanner™ (Amersham Biosciences) and annotated with Microsoft PowerPoint. Probes corresponding to nucleotides +1 through +1848 on the *AGT1 *ORF, -73 through +1845 of the *MAL31 *gene, or +77 through + 1333 of the *SUC2 *locus were generated by PCR using primers AGT1-F and AGT1-R, MAL31-F and MAL31-R, and SUC2-F and SUC2-R (Table 1), respectively.

### Analytical methods

Sucrose was quantified using 50 U/mL of yeast β-D-fructosidase (Sigma) in 50 mM citrate-phosphate buffer, pH 4.5, followed by glucose determination. Glucose was measured by the glucose oxidase and peroxidase method using a commercial kit (BioDiagnostica-Laborclin). Maltose was determined spectrophotometrically at 540 nm with methylamine in 0.25 M NaOH as described previously [50]. Ethanol was determined with alcohol oxidase and peroxidase as described previously [44,50]. Cellular growth was followed by turbidity measurements at 570 nm after appropriate dilution, and yeast cell dry weight was determined as described elsewhere [77]. Briefly, from 1 to 5 mL of fermentation broth was filtered through pre-weighed filters (0.45 μm mixed nitrocellulose and cellulose acetate filters), washed twice with 5 mL of distilled water, and after placing in a small (5 cm diameter) covered Petri dish, dried for 3 to 5 min in a microwave oven at maximum power (900 W) until constant weight. The sugar consumption rates were calculated using samples harvested from the logarithmic growth phase and/or in intervals during which maximal rates were attained. Mean values of dry weight in the specified time intervals were used in the rate calculations. Specific growth rate (μ, h^-1^) was determined as slope of a straight line between ln OD_570 nm _and time (h) during the initial (~12 h) exponential phase of growth. Biomass and ethanol yield coefficients (*Y*_x/s _and *Y*_e/s_, respectively) were obtained at the end of cell growth or ethanol production, taking into account the amount of sugar utilized. The kinetics of active H^+^-sucrose or H^+^-maltose symport were determined as previously described [40,41] using a PHM84 research pH meter attached to a TT1 Servograph (Radiometer, Copenhagen). Initial rates of sugar-induced proton uptake were calculated from the slope of the initial part (< 10 s) of the curve obtained in the recorder, subtracting the basal rate of proton uptake observed before addition of 0.1–100 mM of the sugar. All determinations were done at least in duplicate, and assays were monitored so that no more than 5% of the substrate was depleted. All activities were expressed as nmol of substrate transported per mg dry cell weight per min.

## Competing interests

The author(s) declare that they have no competing interests.

## Authors' contributions

FB, MGD, SLA-J, MLAC, LCM and BUS conceived, designed and performed the experiments. PSDA and BUS contributed with reagents and materials, analyzed the data, and wrote the paper. All authors read and approved the final version of the manuscript.
